# Sickness Presenteeism Among the Swedish Self-Employed During the Covid-19 Pandemic

**DOI:** 10.3389/fpsyg.2021.723036

**Published:** 2021-09-20

**Authors:** Stig Vinberg, Bodil J. Landstad, Åsa Tjulin, Mikael Nordenmark

**Affiliations:** ^1^Department of Health Sciences, Faculty of Human Sciences, Mid Sweden University, Östersund, Sweden; ^2^Nord-Trødelag Hospital Trust, Levanger, Norway; ^3^Unit of Research, Education and Development, Östersund Hospital, Östersund, Sweden

**Keywords:** sickness presenteeism, self-employed, COVID-19, working conditions, business operations, well-being, work-life balance, presenteeism reasons

## Abstract

The present study analyzed the impact of business operations, work and family circumstances, and well-being on the risk of sickness presenteeism for Swedish self-employed workers during the Covid-19 pandemic. It is of great importance to investigate the impact of the pandemic on the self-employed and their enterprises because they are seen as key drivers of economic growth and constitute an expanding group in many countries. Data were obtained from 845 self-employed workers by a web-based survey including questions about background information, work and family circumstances, well-being, sickness presenteeism, and questions about the pandemic. Results were that around 40% of the self-employed introduced new products, processes, and marketing methods, and just over 50% attempted to get new customers during the pandemic. Nearly half of the self-employed people reported that they lost contracts, and 22% judged the risk of bankruptcy to be quite or highly likely. Regression analyses showed that the more the self-employed reported impact on business indicators, increased work hours, a higher level of work-family conflict, and a lower level of mental well-being, the higher the risk of sickness presenteeism. The most common reasons given by the participants for sickness presenteeism during the pandemic were “nobody else can carry out my responsibilities,” “I can't afford to take sick leave” and “I enjoy my work.” Conclusions are that a critical event such as the pandemic probably adds to an already high workload for the self-employed. Impact on business operations such as developing new products/services and marketing, risk of bankruptcy and increased work hours seems to be important factors for explaining sickness presenteeism among the self-employed. Theoretical contributions from the study suggest that critical events such as the Covid-19 pandemic should be considered as an important environmental factor when studying sickness presenteeism among self-employed.

## Introduction

The Covid-19 pandemic has brought about the largest global economic crisis in modern working life (Blundell and Machin, 2020). One of the responses to the pandemic in many countries has been extensive governmental actions to assist the self-employed. These include income protection, expansion of paid sick leave, adjustment support, and financial turnover support (Tetlow and Dalton, 2020). In Sweden, support for businesses has primarily centered on central government schemes to subsidize rent for those enterprises most affected by the crisis (Tetlow and Dalton, 2020). Other business support in Sweden has come in the form of social security contributions, income support measures for individuals and households, tax deferrals, bank loans for micro- and small-sized enterprises, capital injections in strategically important companies and support for the start-up of micro-sized enterprises (Tetlow and Dalton, 2020). However, many self-employed people have not sought governmental support because they perceive that they do not fulfill the roles for applications or they are not sure whether they are eligible (Blundell and Machin, 2020; Eib and Berhard-Oettel, 2020). It is important to understand the impact of the pandemic on the self-employed and their enterprises because they are seen as key drivers of economic growth and constitute an expanding group in many countries (Eurofound, 2017). Some 15% of the European labor market is comprised of the self-employed, with an increase in the share of self-employed people that do not have any employees (Eurofound, 2017). In Sweden, the number of self-employed people (including enterprises with and without employees) is around 96% of the total number of enterprises (Swedish Agency for Growth Policy Analysis, 2019).

A large portion of the self-employed are likely to have been heavily hit by the pandemic because they often have fewer in-house resources (personnel, human resources, and economic) compared to large enterprises, they face a high risk of income loss, and they have difficulties working with customers due to restrictions on mobility (Shafi et al., 2020; Stephan et al., 2020a). Recent studies during the first phase of the crisis show that European self-employed reported significantly higher job insecurity and a worse domestic financial situation compared to employees (Eurofound, 2020a). In addition, the reduction in hours and income for the self-employed contributed to a deterioration of subjective well-being compared to waged workers (Yue and Cowling, 2021). It is likely that reduction in work hours and income for the self-employed are a consequence of societal restrictions, which negatively influence their customer relations. Around 50% of the Swedish self-employed reported a deterioration in the profitability of their businesses due to reduced demands for their products and services, and problems with the supply chain and reaching customers (Salesforce, 2021). Another study from the first phase of the pandemic showed lower scores given for well-being among Swedish self-employed people compared to scores prior to the pandemic (Eib and Berhard-Oettel, 2020). A mixed-method study of managers in Swedish micro-sized enterprises, which are common among the self-employed, showed significantly worse scores for well-being outcomes compared to small-sized enterprises. The study also showed that the managers reported increased workload with extended work tasks during the pandemic (Vinberg and Danielsson, 2021). However, it is important to remember that the self-employed are a diverse group with some becoming more profitable during the pandemic due to increased demand for their products and services (Blundell and Machin, 2020).

For the self-employed, sickness presenteeism (or presenteeism) (SP) is a current phenomenon related to well-being and health outcomes. SP refers to “the phenomena of people turning up for work despite medical complaints and ill-health that would normally require rest and absence from work” (Aronsson and Gustafsson, 2005). SP is important because it can negatively impact both individuals' health (Skagen and Collins, 2016) and organizational productivity (Johns, 2010). Research shows that employees who go to work when ill tend to commit errors more frequently (Niven and Ciborowska, 2015) and report lower levels of performance and productivity (Robertson and Cooper, 2011). Studies in the United States indicate greater losses in productivity and higher costs for SP than for sickness absenteeism (Collins et al., 2005). Another study of the macro-economic impact of presenteeism showed that the annual cost of presenteeism to the Australian economy was estimated to be nearly four times the cost of absenteeism (Econtech, 2008). Concerning small enterprises and the self-employed, the economic consequences of SP may be experienced more acutely than in larger organizations due to the size and structure of the enterprises (Cocker et al., 2013).

Research shows that the self-employed have a high pace of work and work many and irregular hours, indicating that it can be problematic and frustrating for them to stay at home due of illness (Nordenmark et al., 2019). In addition, the self-employed can be viewed as a group that has low replaceability, which can lead to high SP (Kinman and Wray, 2018). It can be assumed that the outbreak of Covid-19 added to an already high workload for self-employed people. According to Knani et al. (2018), SP in small enterprises, where the self-employed often work, remains understudied. In addition, research points to the need for more studies concerning presenteeism related to specific contextual factors such as occupational groups and their working conditions (Ruhle et al., 2020) and environmental factors at a societal level (Lohaus and Habermann, 2019).

The aim of this study was 2-fold. First, we aimed to analyze whether the impact on business indicators, work and family circumstances, and well-being has increased the risk of SP for Swedish self-employed workers during the Covid-19 pandemic. The second aim was to investigate reasons for SP in this group during the pandemic.

## Background

### Work-Family Circumstances and Well-Being Among the Self-Employed

The majority of self-employed people are either sole traders, such as independent contractors (Gallagher and Sverke, 2005), or have micro-sized (up to 10 employees) and small (up to 50 employees) businesses. Research into working conditions for the self-employed shows that they often are exposed to demanding psychosocial working conditions, high levels of pressure, high work demands, many responsibilities, and long and irregular working hours (Nordenmark et al., 2012; Legg et al., 2015; Hagqvist et al., 2016; Stephan, 2018). However they have high job control and the freedom to decide what work tasks to do and how to perform them (Stephan and Roesler, 2010; Nordenmark et al., 2012; Stephan, 2018). Some researchers characterize the work of the self-employed as “active jobs” (Karasek and Theorell, 1990; Stephan, 2018) entailing a combination of high work demands and high job control. The majority of European self-employed workers, with and without employees, report that they have a high level of work quality and well-being, but around one fifth report that they are self-employed out of necessity with little autonomy, and a worse level of work quality and well-being (Eurofound, 2017). This heterogeneity is confirmed by another study of European self-employed workers, which identified distinct profiles among the self-employed that were associated with significant differences in work-related variables and well-being (Bujacz et al., 2020).

A large number of studies have verified that self-employed people are healthier, happier, and more satisfied at work than employed workers (e.g., Andersson, 2008; Stephan and Roesler, 2010; Sevä Johansson et al., 2016). Reasons suggested for these results are that the self-employed have high levels of autonomy and flexibility, and a strong feeling of pursuing their goals (Shir et al., 2019). Other reasons suggested by some researchers are related to selection bias aspects, that particular types of individuals are more likely than others to pursue self-employment, for example stress-resistant individuals (Stephan et al., 2020b). However, a study in the United Kingdom showed that individuals with poorer mental health were more likely to change from employment to self-employment (Stephan et al., 2020a). Other studies indicate that the self-employed have worse well-being (e.g., Parslow et al., 2004; Gunnarsson et al., 2007) or that there are no differences in well-being compared to organizational employees (Andersson, 2008). According to Stephan (2018), high uncertainty, great responsibility for their businesses and employees, and time pressure over longer periods can result in mental and physical disorders. Mental health and well-being are important for the self-employed because research shows they are associated with organizational performance and entrepreneurship (Wincent et al., 2008).

In terms of issues outside work, many studies into work-life balance show that work has a greater negative impact on the private lives of self-employed people (with and without employees) compared to organizational employees (Bunk et al., 2012; Sevä Johansson and Öun, 2015; Annink et al., 2016; Hagqvist et al., 2016). Although self-employed people report having more autonomy in their work than employees do, they also experience greater conflict between work and family life and lower satisfaction with family life (Parasuraman and Simmers, 2001). However, research also indicates that self-employed people are able to manage the competing demands of work and family (Prottas and Thompson, 2006; Sevä Johansson and Öun, 2015) through work flexibility (Prottas and Thompson, 2006).

### Sickness Presenteeism in General and Among the Self-Employed

SP is an important factor in health and well-being given the assumption that it is problematic for self-employed people to stay at home when they are sick as nobody else can do their jobs. Comprehensive research shows that a large number of individual and organizational factors can cause SP. Investigations into SP have been criticized for their limited theoretical approaches (Johns, 2010). However, during recent years some models have been developed with key variables associated with SP. In one model, Johns (2010) classified potential determinants of SP into factors related to organizational policies (e.g., sick pay and attendance control), job design (e.g., job demands, ease of replacement and teamwork), and presenteeism cultures (e.g., SP attitudes). The results of a meta-analysis of significant causes of SP (Miraglia and Johns, 2016), showed that there were prominent correlates with general ill health, job insecurity, job demands, stress, lack of job and personal resources, negative relational experiences, and positive attitudes. These researchers proposed a conceptual model including absenteeism constraints, job demands, job resources, and personal resources as factors that directly or indirectly influence SP. Lohaus and Habermann (2019) developed a similar model that consisted of personal, work-related, and organizational variables. However, they also introduced environmental factors into the model such as a poor economic climate and organizational downsizing. According to a systematic review of longitudinal studies, most studies found that SP at baseline was a risk factor for future sickness absence and decreased self-rated health. However, the findings highlight that no consensus has yet been reached in terms of physical and mental health (Skagen and Collins, 2016).

Work factors such as job demands and job control are significantly related to SP. Job demand factors can be grouped into role demands (e.g., role ambiguity, heavy workload, and supervisory duties), time demands (e.g., overtime, time pressure, and long working hours), and global or overall demands (Miraglia and Johns, 2016). Several studies have found positive associations between these factors and SP (e.g., Hansen and Andersen, 2008; Kinman and Wray, 2018). In addition, financial pressures and job insecurity have also been shown to be important reasons for individuals working despite being ill (Karanika-Murray and Cooper, 2018). When it comes to the self-employed, it is likely that job demand factors are of particular importance since research shows that they have a high working pace and work long hours (e.g., Nordenmark et al., 2012; Hagqvist et al., 2016). In a study of European self-employed workers (Nordenmark et al., 2019), results showed that the self-employed reported a higher level of SP than employees, and that indicators of time demands (working hours, work in the evenings, and work in free time) were significantly associated with the risk of SP. This result is in line with a study showing that self-employed people were more likely to exhibit SP than paid workers, and that working condition variables in particular seemed to affect SP among self-employed workers (Kim et al., 2014). A Danish study by Hansen and Andersen (2008) confirmed that there was a higher risk of SP among self-employed people than employees.

Although job control is considered to have a weaker correlation to SP than job demands, some studies show that job control and SP are related. For example, Biron and Saksvik (2009) found that a lack of control was a determinant of SP. Other factors that are relevant to SP among the self-employed are personal resources, different health outcomes, optimism, conscientiousness, work engagement, and job satisfaction (Miraglia and Johns, 2016). Job satisfaction and work engagement have been shown to be a predictor of SP, although not all studies support a positive relation to SP (Karanika-Murray and Cooper, 2018). Difficulties in finding replacement staff has been shown to be associated with higher levels of SP (Aronsson and Gustafsson, 2005; Widera et al., 2010).

Work-family conflict has been shown to be positively associated with SP (Miraglia and Johns, 2016). Work-family conflict may be a symptom of excessive workload or long working hours, which may cause conflict at home as employees may need to take work home and thus reduce family time, and/or force attendance at work even when sick (Miraglia and Johns, 2018). Some studies have also shown that family-work conflict are positively related to SP (Miraglia and Johns, 2016). In summary, extensive research shows that on the one hand, the self-employed have demanding psychosocial working conditions, but on the other hand, they have great job control and flexibility in their work. Most studies indicate that the self-employed have better self-rated health and life satisfaction compared to employees. However, research results are contradictory in this field. One explanation behind these results might be the differences in motives for self-employment, sector, and company size amongst the self-employed. Proposed models for studying SP include a large number of variables related to individual-, work-, organizational- and environmental factors of relevance for the self-employed. Multiple levels of determinants of SP operate together rather than in isolation, and it seems that work-related factors are particularly important in determining individuals' decisions to go to work while ill (Karanika-Murray and Cooper, 2018). A comprehensive review that integrated 109 samples including nearly 17 000 participants and 55 variables reported on the associations between determinant variables and SP (Miraglia and Johns, 2016). They found positive correlations of presenteeism with several variables of relevance to our study; e.g., absenteeism, personal financial difficulties, job insecurity, workload, time demands, work hours, work-family conflict, and job satisfaction. Examples of negative correlations were also found, for instance health and ease of replacement.

Based on the above-described theoretical aspects of determinants associated with SP our study focuses on individual, work, organizational and environment-related variables (Lohaus and Habermann, 2019). In our study, job satisfaction, work-family conflict, family-work conflict, mental well-being, and sickness absenteeism can be seen as individual-related variables. Work-related variables includes business indicators and an increase in work hours as work-demand factors. In addition, the business indicators used (loss of contract and risk of bankruptcy) can be seen as related to organizational variables (e.g., job insecurity and under-staffing) and environmental variables (e.g., economic climate). The focus of the present study is to consider business indicators as work demand factors among the self-employed, and to investigate their association with SP when controlling for indicators of work-family circumstances, well-being and background characteristic variables.

## Materials and Methods

### Data Sources and Sample Selection

The quantitative methodology used in this research was based on an e-survey used by Eurofound to capture the immediate impact of the Covid-19 pandemic on the way people in Europe live and work (Eurofound, 2020a). Most of the questions are based on Eurofound's European Quality of Life Survey (EQLS) and European Working Conditions Survey (EWCS), while some questions are new. The EQLS and EWCS use validated questions and thorough procedures for questionnaire construction, sampling and interviewing when comparing individuals in European countries (Eurofound, 2020b). Permission has been granted for us to use these questions. Additional questions concerning reasons for SP have been used in other studies in Norway and Sweden (Hansen and Andersen, 2008). The questionnaire consisted of 76 questions divided into four clearly differentiated blocks including background information, working conditions, work-life balance, and well-being, as well as questions about the Covid-19 pandemic. We used a panel platform (Cint) that included different sub-panels related to occupational groups (owners/managers in small companies in our study) provided by Netigate, an organization specialized on on-line research (https://Netigate.net/). The survey was distributed between 18 March and 12 April 2021 to self-employed people in companies with fewer than 50 employees. The sample is a non-probability sample, however it was selected based on the characteristics of self-employed people and based on the objective to study self-employed people in companies with <50 employees. The self-employed represented eight common sectors [agriculture (7%), industrial manufacturing (9%), construction (15%), transport (11%), finance (15%), retail (23%), education (9%), and health (11%)] in the Swedish small-business labor market, and almost all Swedish regions were included. According to the Swedish Agency for Growth Policy Analysis (2018), the most common sectors among the Swedish self-employed are agriculture, industrial manufacturing, retail and the service sectors, such as finance, education, and health.

The total sample group consisted of 845 self-employed workers including owners (62%) and/or CEOs/managers (22%) and, in some cases, those who combine business with employment (16%). After removing incomplete surveys, the final sample consisted of 814 self-employed workers.

### Indicators and Variables

Based on the survey questions, variables were established for indicators of business, work and family circumstances, and well-being. *Sickness presenteeism* was used as an outcome variable and measured by the single-item question “During the last 12 months have you worked even though you were sick?” The response alternatives were 1 or 2 (1 = no, 2 = yes).

The index for the *Impact on business operations* included four questions about whether new or changed products, processes or marketing methods had been introduced, or whether efforts had been made to find new customers. The scale was 1 or 2 (1 = no, 2 = yes) and Cronbach's alpha was 0.72. *Risk of bankruptcy* was measured by the single-item question “How likely or unlikely is it that your business will go bankrupt within 3 months?” The scale was 1–5 (1 = very unlikely, 2 = quite unlikely, 3 = neither likely nor unlikely, 4 = quite likely, 5 = highly likely). *Loss of contracts* was measured by the single-item question “During the Covid-19 pandemic have you lost your job(s)/any contract(s)?” The scale was 1 or 2 (1 = no, 2 = Yes, permanently or temporarily). *Increase in work hours* was measured by the single-item question “During the Covid-19 pandemic have your working hours…?” The scale was 1–5 (1 = decreased a lot, 5 = increased a lot). *Job satisfaction* was measured by the question “In general, are you satisfied, not particularly satisfied, or not at all satisfied with your working conditions?” The scale was 1–4 (1 = not at all satisfied, 4 = very satisfied). The index for *Work-family conflict* included three questions about the extent of worry about work after the working day, whether tiredness after work hinders housework and whether work reduces time for family activities. The scale was 1–5 (1 = never, 5 = always) and Cronbach's alpha was 0.77. The index for *Family-work conflict* included two questions about difficulties in concentrating on work because of family responsibilities and family responsibilities preventing time for work. The scale was 1–5 (1 = never, 5 = always) and Cronbach's alpha was 0. 81. *Mental well-being* consisted of five items including whether the respondent felt calm and relaxed, felt happy and positive, felt active and energetic, felt fresh and rested, and that life was of interest over the last 2 weeks. The mental well-being index had a 6-point response scale (1 = never, 6 = all the time) and the calculated Cronbach's alpha was 0.71. Mental well-being is a broad concept widely studied by the World Health Organization's Well-Being Index (WHO-5), a 5-item index assessing subjective psychological well-being (Topp et al., 2015). *Sickness absenteeism* was measured by the single-item question “How many days have you been away from work during the last 12 months due to sick leave or health-related absence?” The variable was constructed as 1–2 (1 = 1–7 days, 2 = more than 7 days) in accordance with other studies (Taloyan et al., 2012). The background factors used were *age* (years), *gender* (1 = man, 2 = women), *level of education* (1 = compulsory or 9 years, 2 = upper secondary school or 12 years of education, 3 = University education) and company size (1= 0 employees, 2 = 1–9 employees, 3 = 10–19 employees, 4 = 20–49 employees).

### Statistical Analyses

A cross-sectional study was conducted. Statistical analyses consisted of descriptive statistics for background variable data calculated using means and percentages. For variables related to indicators of business, work and family circumstances, and well-being, numbers and percentages were calculated. For indices measuring impact on business operations, work-family conflict, family-work conflict, and mental well-being, Cronbach's alpha values were computed in order to estimate the internal reliability. Correlation coefficients between the variables were analyzed using the Pearson correlation coefficient. Logistic regression analyses were carried out in four phases. Logistic regression is an appropriate method to use when the dependent variable is dichotomous, which is the case in this study. The reason for performing the analyses in four separate models is that it makes it possible to control for different categories of variables in different steps. Model 1 shows the result of an analysis of the relationship between business indicators and the risk of SP. Model 2 controlled for indicators of work and family circumstances, Model 3 controlled for both indicators of work and family circumstances and well-being, and finally, Model 4 included background characteristics variables. Odds ratios (ORs) are presented as measures of the relative risk of SP. All statistical analyses were carried out using IBM SPSS Statistics 27.

## Results

### Descriptive Statistics

Of all the self-employed included in the study, 38% were women, the mean age was 41.2 years, 53% had a University education, 66% were married or cohabitated, and 47% had children living at home. The business size distribution was 0 employees (30%), 1–9 employees (45%), 10–19 employees (17%), and 20–49 employees (8%).

Table 1 presents descriptive data for the indicators and variables used. Around 40% of the self-employed introduced new products, processes, and marketing methods, and just over 50% attempted to get new customers during the pandemic. Hardly 50% of self-employed people reported that they lost contracts, and 22% judged the risk of bankruptcy within 3 months to be quite or highly likely. Nearly one third of the self-employed experienced an increase in work hours.

**Table 1:** Descriptive data of study variables (*N* = 814).

**Business indicators**	***n* (%)**	***N***
**Impact on business operations**		
- New products or services have been introduced (yes)	296 (39)	760
- New processes have been introduced (yes)	318 (42)	760
- New marketing methods have been introduced (yes)	270 (36)	760
- Attempt to get new customers (yes)	407 (54)	760
Loss of contract (yes)	365 (47)	781
Risk of bankruptcy		759
- Highly likely	74 (10)	
- Quite likely	90 (12)	
- Neither likely or unlikely	185 (24)	
- Quite unlikely	115 (15)	
- Very unlikely	295 (39)	
**Indicators of work-family circumstances**		
Job satisfaction (satisfied/very satisfied)	592 (76)	781
Increase in work hours (somewhat/a lot)	227 (29)	781
Work-family conflict		776
- Worry about work after the working day (always/most of the time)	297 (38)	
- Tiredness after work hinders housework (always/most of the time)	212 (27)	
- Work reduces time for family activities (always/most of the time)	165 (21)	
Family-work conflict		776
- Difficulties concentrating at work due to family responsibilities (always/most of the time)	139 (18)	
- Family responsibilities hinder time at work (always/most of the time)	163 (21)	
**Indicators of well-being**		
Mental well-being		768
- Felt calm and relaxed	293 (38)	
- Felt happy and positive (always/most of the time)	262 (34)	
- Felt active and energetic (always/most of the time)	225 (29)	
- Felt fresh and rested (always/most of the time)	214 (28)	
- Daily life consists of interesting things (always/most of the time)	287 (37)	
Sickness absenteeism (> 7 days)	203 (27)	761
Sickness presenteeism (yes)	337 (44)	761

*Internal failures of 33–55 for different questions*.

Three out of four self-employed people reported that they were satisfied or very satisfied with their jobs. When it comes to work-family conflict variables, the highest score was reported for the question on worry about work after the working day (38%). For family-work conflict questions, the highest score was reported for the question on family responsibilities hinder time at work (21%). Around one quarter (27%) of the self-employed reported sickness absenteeism of more than 7 days, and around one third of self-employed workers gave high scores for questions related to mental well-being. Around four out of ten self-employed people reported that they had experienced SP, i.e., that they had worked despite being sick during the last 12 months.

The first two columns in Table 2 present the means and standard deviations for all study variables used. The correlations between SP and variables for business indicators, and indicators of work and family circumstances and well-being are all significant in the expected direction. The higher the values for impact on business operations, loss of contract, risk of bankruptcy, and increase of work hours, the higher the risk for SP. Correlations were highest between work-family conflict and family-work conflict (0.80) and between loss of contract and risk of bankruptcy (0.50). There was no significant relationship between SP and age, gender, and education of the self-employed worker. There was also no significant relationship between SP and company size (not shown in the table). The correlations were not sufficiently strong to suspect multi-collinearity, which would be the case if the correlation coefficients were ~0.8 or higher (Shrestha, 2020).

**Table 2:** Means, standard deviations, and correlations (Pearson) between sickness presenteeism and indicators of business, work and family and well-being.

	**M**	**SD**	**1**	**2**	**3**	**4**	**5**	**6**	**7**	**8**	**9**
1. Sickness presenteeism	0.44	0.49									
2. Impact on business operations	1.42	0.36	**0.25**								
3. Loss of contract	1.47	0.50	**0.20**	**0.40**							
4. Risk of bankruptcy	2.38	1.36	**0.27**	**0.44**	**0.50**						
5. Job satisfaction	2.99	0.82	**−0.09**	**−0.09**	**−0.28**	**−0.24**					
6. Increase in work hours	2.93	0.82	**0.22**	**0.15**	–0.05	**0.20**	0.02				
7. Work-family conflict	2.94	0.98	**0.28**	**0.41**	**0.32**	**0.41**	**−0.14**	**0.10**			
8. Family-work conflict	2.60	1.04	**0.24**	**0.33**	**0.28**	**0.40**	**−0.10**	**0.16**	**0.80**		
9. Mental well-being	3.76	1.11	**−0.12**	–0.04	**−0.15**	**−0.13**	**0.39**	**0.08**	**−0.31**	**−0.18**	
10. Sickness absenteeism	11.8	36.4	**0.08**	**0.13**	**0.17**	**0.18**	**−0.14**	–0.06	**0.13**	**0.12**	**−0.08**

*Figures in bold: p < 0.05*.

To analyze the risk of SP among the self-employed, multiple regression were carried out to estimate the odds ratios (OR) for variables related to business, work and family circumstances, and well-being indicators. Model 1 in Table 3 shows that the variables impact on business operations (OR = 2.41) and risk of bankruptcy (OR = 1.32) are significantly associated with SP. The more that self-employed workers dealt with implementing new products, services, processes, and marketing, and made efforts to get new customers, the higher risk of SP. In addition, the more they perceived that there was a risk of bankruptcy, the higher the risk of SP. When controlling for variables related to indicators of work and family circumstances in model 2, the variables impact on business operations, risk of bankruptcy, increase in work hours, and work-family conflict were significantly related to a higher risk of SP. The same pattern was present in model 3, wherein mental well-being was also significantly related to a lower risk of SP.

**Table 3:** Logistic regression.

**Independent variables**	**Model 1**	**Model 2**	**Model 3**	**Model 4**
Constant	0.082	0.021	0.032	0.015
**Business indicators**				
Impact on business operations	2.416^***^	1.680^*^	1.753^*^	1.739^*^
Loss of contract	1.246	1.383 (^*^)	1.369 (^*^)	1.410 (^*^)
Risk of bankruptcy	1.324^***^	1.160^*^	1.155^*^	1.149 (^*^)
**Indicators of work-family circumstances**				
Job satisfaction		0.960	1.028	1.043
Increase in work hours		1.400^***^	1.421^***^	1.406^***^
Work-family conflict		1.556^**^	1.442^*^	1.448^*^
Family-work conflict		0.948	0.968	0.981
**Indicators of well-being**				
Mental well-being			0.860 (^*^)	0.858 (^*^)
Sickness absenteeism			1.173	1.187
**Background characteristics**				
Age				1.000
Gender				0.996
Education				1.191
Company size				1.105
Nagelkerke R square	0.125	0.193	0.199	0.210

*Indicators of business factors, work-family factors, well-being factors and background characteristics by sickness presenteeism (Odds Ratios)*.

*** *p < 0.001*,

** *p < 0.01*,

* *p < 0.05*,

(^*^) p < 0.10.

Model 4 also included variables related to background characteristics. In this phase of the analysis the variables impact on business operations (OR = 1.74), loss of contract (OR = 1.41), risk of bankruptcy (OR = 1.15), increase in work hours (OR = 1.41), work-family conflict (OR = 1.45), and mental well-being (OR = 0.86) were significantly related to a higher risk of SP. Therefore, the more the self-employed reported impact on business indicators, increased work hours, a higher level of work-family conflict, and a lower level of mental well-being, the higher the risk of SP. The variable sickness absenteeism was not significantly associated with SP. None of the background characteristic variables were significantly related to SP. The Nagelkerke R-squared in the final model was 0.21.

Table 4 shows that the most common reasons given by the participants for SP during the pandemic were “nobody else can carry out my responsibilities,” “I can't afford to take sick leave,” and “I enjoy my work.”

**Table 4:** Description of reasons for presenteeism among the self-employed (*n* = 337).

**Reasons**	***n***	**%**
I do not want to burden my colleagues	80	23.7
Nobody else can carry out my responsibilities	198	58.7
I enjoy my work	132	39.2
I can't afford to take sick leave	140	41.5
I do not want to be considered lazy	65	19.3
I am too pride to take sick leave	77	22.8
I do not want to be suspected of cheating	36	10.7
Going to work was beneficial for my health	63	18.7
I want to maintain my social network	38	11.2
I am worried about losing my job	45	13.4

*Reasons for presenteeism add up to more than 100%, because several reasons could be selected*.

## Discussion

The aim of this study was to contribute to knowledge about how business, work and family circumstances, and well-being indicators have increased the risk of SP among the Swedish self-employed during the Covid-19 pandemic. The main result was that the business indicators were significantly associated with SP, even when controlling for indicators of work-family circumstances, well-being and background indicators. In addition, the variables increase in work hours and work-family conflict were significantly associated with SP. When self-employed workers also reported that a main reason for SP was that no one else could do their job, it is likely that the pandemic has added to an already high workload, which has increased the risk of SP.

Extensive research shows that SP in individuals is a risk factor for future deterioration of health and loss of productivity in organizations (Johns, 2010; Ruhle et al., 2020). It is of great relevance to study the self-employed in relation to the pandemic because of their relevance in working life, and because studies have shown that this group may be negatively affected by the pandemic in several ways. The pandemic might influence their businesses negatively resulting in income loss, and lead to increased workload and worse well-being. Due to the low replaceability of the self-employed, SP is a prevalent health-related outcome in this group and there is a need for more studies of SP among the self-employed. SP is a particular challenge for the self-employed, who most often work in small companies where the personal and economic consequences of SP can be more acutely felt than in larger enterprises (Cocker et al., 2013).

The results of this study show that 44% of self-employed workers reported SP during the last year, which is slightly lower than a study of European self-employed people in which 52% reported SP (Nordenmark et al., 2019) and another study of different occupational groups in Sweden in which 56% reported SP (Johansen et al., 2014). One explanation behind this result might be that in our study the mean age was relatively low, and the number of male self-employed workers was relatively high. Therefore, this comparison must be made with caution. Participants indicate that the main reason for SP is that “nobody else can carry out my responsibilities.” This is in line with another study of SP in Norway and Sweden, which found that among the self-employed, the factor “nobody else can carry out my responsibilities” was significantly related to SP in regression analyses (Johansen et al., 2014). Research has also shown that low replaceability is a factor related to SP for individuals in leading positions (Aronsson and Marklund, 2018).

The fact that the variables impact on business indicators and increase in work hours during the pandemic are significantly associated with SP in the final regression model can be an expression of increased work tasks and workload for the self-employed. In addition, the variable risk of bankruptcy may lead to self-employed people increasing their efforts to handle different work tasks. This result is in line with earlier research showing that job demand factors are important predictors of SP (Miraglia and Johns, 2016; Lohaus and Habermann, 2019; Ruhle et al., 2020). Extensive research shows that a wide range of job demands and stress-related features at the workplace increase the occurrence of SP (Miraglia and Johns, 2016). Factors such as a heavy workload, understaffing, and overtime are prominent correlates that can contribute to ill-health, which can be seen as a mediating factor between negative workplace features and SP (Pohling et al., 2016). A study of the self-employed in Northwestern Europe (Nordenmark et al., 2019) confirms that time-demand factors, including the level of working hours, work in the evenings, and work in free time, are predictors of SP. It is likely that the pandemic has led to a high workload and concern about business survival among the participants, which has also contributed to SP.

The fact that work-family conflict is significantly associated with SP in the regression analyses is in line with earlier research (Miraglia and Johns, 2016). Work-family conflict may be an expression of a high level of workload and long work hours, which can cause conflict at home because it reduces time for family activities (Miraglia and Johns, 2018). According to Schjoedt (2021), long work hours and coping with the challenges of starting and managing a business can lead to work-family conflict for the self-employed. The finding that family-work conflict is not significantly associated with SP is not in line with earlier research (Miraglia and Johns, 2016).

The results showing that the determinant variable mental well-being reduces the occurrence of SP (with a *p*-level <0.10) are in accordance with earlier research that shows relationships between SP and different physical and mental health outcomes, and risks of future ill health and future absenteeism (e.g., Aronsson and Gustafsson, 2005; Bergström et al., 2009; Gustafsson and Marklund, 2011; Miraglia and Johns, 2018; Ruhle et al., 2020). The results in the final regression model showing that the background variables age, gender, education, and company size do not contribute significantly, are supported by some earlier studies showing that these variables have low explanatory values for SP (Aronsson and Marklund, 2018).

Although, research into the positive consequences of SP is limited, during recent years researchers have identified positive consequences for individuals and organizations (e.g., Karanika-Murray and Biron, 2020). For instance, SP can be positive for individuals in that being committed to work can shift their attention away from illness (Miraglia and Johns, 2018). In a study investigating the positive consequences of SP (Lohaus et al., 2021), significant positive associations were found between SP and variables related to economic orientation, financial advantages, and participants' perception that their health benefited from working. When self-employed workers and their businesses are negatively affected by the pandemic, it is understandable that they are forced to try to find solutions for the company to survive. To that end, SP can be a necessary strategy for work tasks related to governmental financial aid, employee support, and the development of new products and services. Although SP can be positive for the business during a critical event such as the pandemic, it is important for the self-employed to consider the risk of future ill-health.

Established models describing the emergence of SP incorporate variables related to individuals, working conditions, organizational factors, and the environment (e.g., societal, economic, and cultural context aspects) (Miraglia and Johns, 2016; Lohaus and Habermann, 2019). Although several studies have studied SP in different sectors and occupations, there is still a need for more knowledge about the effects of sector-specific work environments on SP. Our study provides theoretical contributions suggesting that critical events such as the Covid-19 pandemic should be considered as an important environmental factor, and that the self-employed constitute an important occupational group related to the individual and work.

## Limitations And Strengths

The individuals included in this study are not part of a randomly selected sample. However, they represent the self-employed within different business sectors and regions in Sweden, in companies with fewer than 50 employees. As the data were cross-sectional, we cannot draw conclusions on causality and causal tendencies. This is perhaps most problematic in terms of the variables measuring work-family conflict and well-being, which are factors that theoretically can be seen as causes of SP as well as consequences of SP. This should be considered when interpreting these results. Measuring SP through a single-item question might also be considered as a limitation, however this measure has been used in other studies measuring SP (Ruhle et al., 2020). One strength of the study is that the survey used has also been used in other European studies (Eurofound, 2020a) with validated questions and indices. The indices in our study show satisfactory Cronbach's alpha values (0.71–0.81). This study contributes to knowledge concerning SP among a major group in working life which is seldom studied in terms of different health outcomes.

## Conclusions And Implications

Several conclusions can be drawn from this study. Many self-employed people were affected by the Covid-19 pandemic in a number of ways that are related to impact on business operations and income loss. It is likely that a critical event such as the pandemic adds to an already high workload for the self-employed with many work tasks. Impact on business operations such as developing new products/services and marketing, risk of bankruptcy, increased work hours and work-family conflict appear to be important factors for explaining SP among the self-employed. Self-employed people report that low replaceability is the main reason for their decision to work even though they are ill, and it is likely that a critical event such as the pandemic forces them into SP for the survival of their businesses.

The results of the study highlight that it is important for the self-employed to receive support for handling SP and their health, as well as extended work tasks related to strategies for developing their businesses. When considering working conditions and health issues, consultants such as those in occupational health services can be a beneficial resource for the self-employed. For business development, governmental bodies and business networks can be valuable for supporting the enterprises to generate ideas about how to find new solutions, products, and services for their businesses. For future research, both qualitative and quantitative longitudinal studies in larger samples of the self-employed in different sectors will be valuable. Future research into SP among the self-employed will need to consider both negative and positive consequences of SP behavior. In addition, there is a need to develop and study individual and workplace-oriented interventions to reduce SP among the self-employed.

## Data Availability Statement

The raw data supporting the conclusions of this article will be made available by the authors, without undue reservation.

## Ethics Statement

The studies involving human participants were reviewed and approved by The Ethics Review Authority (Dnr 2020-05223). The patients/participants provided their written informed consent to participate in this study.

## Author Contributions

SV designed and developed the survey, analyzed the data, wrote the original manuscript, and revised the manuscript. MN provided data analysis ideas, checked the analyses, and revised the manuscript. BL and ÅT contributed to the analyses and writing of the manuscript. All authors contributed to the article and approved the submitted version.

## Funding

This project was funded by AFA Insurance in Sweden, reference number 200235.

## Conflict of Interest

The authors declare that the research was conducted in the absence of any commercial or financial relationships that could be construed as a potential conflict of interest.

## Publisher's Note

All claims expressed in this article are solely those of the authors and do not necessarily represent those of their affiliated organizations, or those of the publisher, the editors and the reviewers. Any product that may be evaluated in this article, or claim that may be made by its manufacturer, is not guaranteed or endorsed by the publisher.
